# Redesigning FDM Platforms for Bio-Printing Applications

**DOI:** 10.3390/mi16020226

**Published:** 2025-02-16

**Authors:** Burak Turker

**Affiliations:** Department of Biomedical Engineering, Engineering Faculty, Ahmet Necdet Sezer Campus, Afyon Kocatepe University, Afyonkarahisar 03200, Turkey; bturker@aku.edu.tr; Tel.: +90-532-6525464

**Keywords:** fused deposition modeling, electrospinning, melt electrowriting, 3D printers, additive manufacturing, bio-printing, modification, design, biomedical applications

## Abstract

Fused Deposition Modeling (FDM) is a prominent additive manufacturing technique known for its ability to provide cost-effective and fast printing solutions. FDM enables the production of computer-aided 3D designs as solid objects at macro scales with high-precision alignment while sacrificing excellent surface smoothness compared to other 3D printing techniques such as SLA (Stereolithography) and SLS (Selective Laser Sintering). Electro-Spinning (ES) is another technique for producing soft-structured nonwoven micro-scale materials, such as nanofibers. However, compared to the FDM technique, it has limited accuracy and sensitivity regarding high-precision alignment. The need for high-precision alignment of micro-scaled soft structures during the printing process raises the question of whether FDM and ES techniques can be combined. Today, the printing technique with such capability is called Melt Electro Writing (MEW), and in practice, it refers to the basic working principle on which bio-printers are based. This paper aims to examine how these two techniques can be combined affordably. Comparatively, it presents output production processes, design components, parameters, and materials used in output production. It discusses the limitations and advantages of such a hybrid platform, specifically from the perspective of engineering design and its biomedical applications.

## 1. Introduction

Additive manufacturing is a term formulated for rapid prototyping methods, popularly known as 3D printing [1]. FDM, developed and produced by Stratasys, USA [2] after being patented in 1992, is the most widely preferred method among additive manufacturing methods thanks to its ability to offer cost-effective, fast solutions. It is one of the widely used extrusion-based additive manufacturing Technologies. It works on the principle of extruding the material to be used for production to the production table in a layered manner through a nozzle [3]. The material is heated in the nozzle to a semi-solid melt [4] and extruded onto the production table to form the desired pattern. As a result of programming the controlled movement of the extruder nozzle or production table in the x-y-z axes [5], 3D production of the desired solid models [6] is realized.

Fibers produced from a polymer solution or polymer melt using various methods in sizes of one micron in diameter and below are called nanofibers [7]. Nanofiber technology can provide solutions to overcome existing challenges in the biomedical field, such as burn and wound care, organ repair, and treatment of various diseases [8]. Electrospinning (ES) is a method for creating polymer-based nanofibers and has been developed to produce nanofibers from polymer solutions [9]. Nanofibers with various morphological properties are formed in a controlled manner by pulling the polymer from the polymer solutions in the electric field created by high voltage [10]. Electrospinning is a simple and versatile technique for producing nanofibers with various morphological properties from different polymer solutions and melts using polymers, metal oxides, and ceramics [11].

Melt Electro Writing (MEW) is a derivative of the electrospinning method and is a 3D printing technology that allows molten polymers to be printed as very fine fibers in a controlled manner. MEW is a remarkable method in tissue engineering and the production of biological materials, especially in biomedical applications. The main difference of this method is that the molten polymers are attracted by electrostatic forces and positioned in a specific and controllable way using a print head [12].

## 2. Features of Fused Deposition Modelling (FDM) Platforms

### 2.1. Design Components and Printing Process

**Figure 1:**
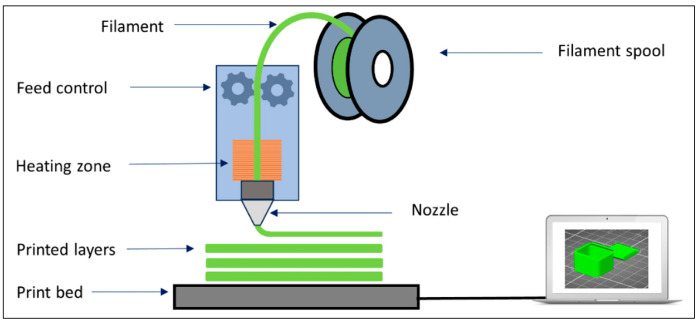
A schematic diagram illustrating the process of an FDM platform (from [13]).

The design components that make up a typical FDM platform are given in Figure 1. It is possible to summarize the basic stages of the output generation process as follows.

- *The semi-solid melt of the material:* The amount of material melted by the heating process in the extruder head’s chamber, the temperature of the molten material, the viscosity of the molten material, and the surface tension vary depending on the feed rate [3].
- *Extrusion of the melt:* Since the geometry of the chamber and nozzle structure significantly affects the behavior and, therefore, the extrusion of the molten material in the chamber, different nozzle designs have been developed [14].
- *Solidification of the material after extrusion:* The ambient temperature must be controlled during solidification to maintain the shape and size of the print [15] under the effects of gravity, surface stress, and cooling [4].
- *Motion control:* With positional path control, the printing process is completed by printing pre-prepared CAD models in layers [16].

### 2.2. Materials Used and Their Biomedical Applications

Although the variety of materials used in the FDM process is increasing day by day, ABS (Acrylonitrile Butadiene Styrene), polylactic acid (PLA), polycarbonate, polyamide, polystyrene, and polyethylene materials with thermoplastic properties are widely used [17]. These materials are used because they have a high level of hardness and strength [18] and a high level of resistance to temperature [19].

Fused Deposition Modeling (FDM) filaments used in biomedical applications need to be made of biocompatible, durable, and sterilizable materials [20]. Such materials can be used in various fields, such as biomedical devices, implants, prosthetics, and biomedical modeling. The properties, usage areas, advantages, and disadvantages of filaments, which are widely used in biomedical applications, can be summarized as follows:

- *PLA* is a biodegradable material, and due to this property, it is frequently used for low-cost prototypes and biomedical modeling. In addition, it can be sterilized and is biodegradable in the body. As such, they are a potential material for temporary implants or tissue engineering [21]. While it is advantageous to be easy to print, environmentally friendly, and biodegradable, its low heat resistance may limit its applications in the biomedical field [22].
- *Polyether Ether Ketone (PEEK)* is a thermoplastic known for its high mechanical properties, chemical resistance, and biocompatibility. For this reason, it is widely used for dental implants, orthopedic implants, and medical devices [23]. In addition, it is suitable for sterilization processes because it is resistant to high temperatures [24]. While it has good mechanical properties, such as high-temperature resistance, biocompatibility, strength, and lightness, it can be considered a disadvantage because it requires high printing temperatures (250–350 °C) and special processing equipment.
- *Nylon* is a durable and flexible material often used in biomedical applications. It is mainly used for prototypes of medical devices, binding elements, and injection parts. It is also used in some types of implants due to its biocompatible properties [25]. While it is a durable, flexible, and chemical-resistant material, it is known as an advantage because it is transparent and cost-effective. At the same time, it has a moisture-absorbing feature and requires processing at high temperatures.
- Thanks to its elastic material properties, *Thermoplastic Elastomer (TPE)* is used in biomedical devices to produce soft, flexible, and comfortable materials [26]. It is especially suitable for prosthetic parts, orthopedic products, and medical devices. In addition to being soft, flexible, and biocompatible, it is safe for health and ideal for food contact [27]. At the same time, due to its elastic properties, the high precision requirement during the printing phase can be considered a disadvantage.
- *Polyvinyl Alcohol (PVA)* supports biomedical models with complex geometries [28]. Since PVA is a water-soluble material, it is often combined with other biomedical filaments (e.g., PLA or ABS) [29]. Its high level of biocompatibility and water dissolution is advantageous for supporting complex models, while its easy exposure to moisture requires careful storage.
- *Metal Composite Filaments* are used for biomedical prototypes and are helpful in the design of implants in some applications [25]. However, these filaments are not used directly in biomedical implants; They are generally preferred for testing and prototyping [23]. Thanks to their durable and strong material structure, they enable the production of genuine metal-looking parts. However, these material properties bring various printing challenges, requiring high-quality and expensive 3D printers.
- *Hydroxyapatite (HA) composite filaments* are used in implants and biomedical models that promote bone healing due to the properties of hydroxyapatite that are similar to bone tissue [30]. These filaments hold potential, especially for tissue engineering and orthopedic implants [31]. Although it is suitable for tissue engineering applications, mainly due to its compatibility with bone tissue and biocompatibility, printing processes have difficulties [32].
- *Bioplastic filaments* are produced from biodegradable materials, are environmentally friendly, and are often used in medical applications [33]. The biocompatibility of bioplastics is also essential, and some are suitable for surgical applications. While eco-friendliness is advantageous due to its biodegradability and compatibility with emerging biotechnologies, some bioplastics may have low mechanical properties [34].

### 2.3. Printing Features and Constraints

Since the production of structurally soft materials is aimed at bio-printing applications, the fact that FDM allows the production of structurally hard materials reveals itself as the main limitation in biomaterial production. Especially in tissue engineering, biocompatible polymers are synthesized in low quantities due to their sufficient use in small amounts and their high cost. This is another fundamental constraint, as FDM requires a high material volume during printing.

Moreover, regarding the strength and hardness level of the materials produced, the necessity of mixing polymers with other materials (such as ceramics) in the FDM technique causes the material to exhibit non-Newtonian behavior, as it seriously affects the flow characteristics [1].

## 3. Features of Electro-Spinning (ES) Platforms

### 3.1. Design Components and Output Generation Process

**Figure 2:**
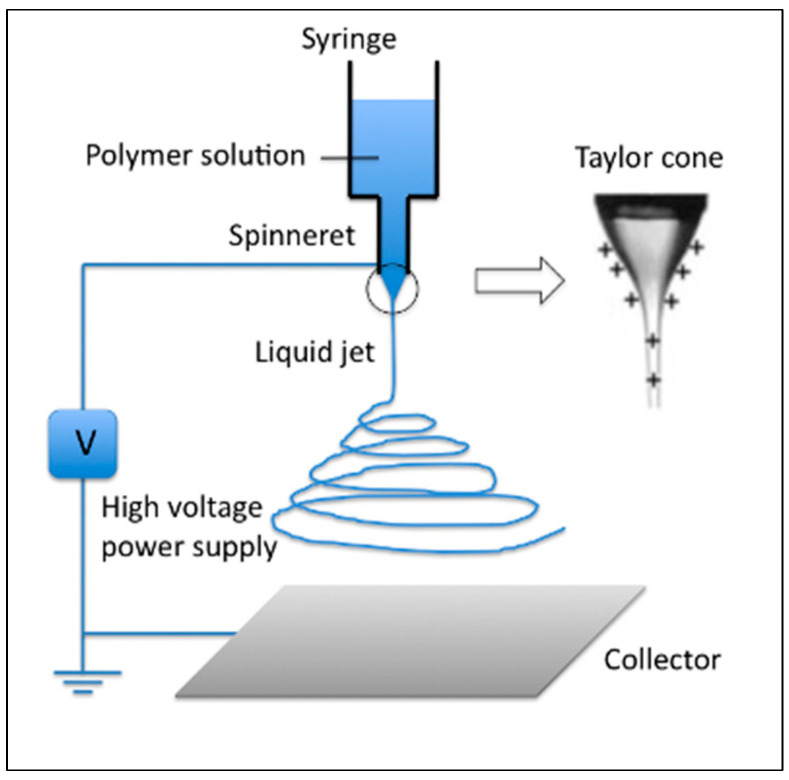
A schematic diagram illustrating the process of an electrospinning platform (from [35]).

The ES method uses a high electric field to produce very thin polymeric fibers with diameters ranging from nanometers to micrometers. The electrospinning mechanism is based on a complex electro-physical activity between the polymer solution and the electrostatic force. The experimental setup required for electrostatic production consists of three critical main parts (Figure 2). The high-voltage power supply, the supply unit (syringe, metal needle, etc.), and the collector (conductive plate, cylinder, etc.) are the most critical components of this mechanism.

Filamentous structures obtained by the electrospinning method can be easily processed due to their small size, large surface area, unique optical properties, and high mechanical strength. This allows nanofibers to quickly enter many industrial areas and find potential uses [36,37]. In the electrospinning method, the polymer is dissolved in a suitable solvent or melted by heat. This polymer is placed inside a glass pipette or syringe with a small hole in the end [38]. Without any electric field, a droplet is formed at the tip of the syringe needle, and the droplet falls due to gravity. In this case, the forces acting on the droplet are the surface tension of the liquid and the force of gravity. A voltage of up to 50 kV is then applied between the polymer solution (or melt) and a collector sheet opposite the open end of the pipette [39]. Suspended at the tip of the needle in the feeder unit, the droplet of polymer solution is spherical in shape due to the forces exerted by the surface tension up to a critical voltage value. When the applied potential difference reaches a threshold value, the electrostatic forces are equalized to the surface tension forces. The polymer droplet changes shape to a Taylor cone form [40]. After the polymer droplet becomes a Taylor cone, a jet [41] gushes out of the cone tip with a minimal increase in voltage. The jet follows different paths as it travels between the collector plate and the metal needle tip. The charged jet [42] moves steadily over a distance after exiting the Taylor cone.

Electrospinning is a technique used to produce nanofibers with many process parameters [43] that need to be considered. The solution is drawn in an electrical field during electrospinning, forming fine fibers. The fibers obtained in this method are used in various fields, especially in medical, textile, filtration, and biotechnology applications [44,45]. A typical electrospinning platform involves several basic process parameters [40]:

- *The parameters for the solution used*, related to its concentration, determine its viscosity and electrical conductivity, significantly affecting the fibers’ formation. A high concentration can produce thicker fibers, while a low concentration produces finer fibers [46]. The fibers’ critical properties are determined by the solvent’s chemical structure and the solution’s composition (polymer type, solvent, etc.). Polymers can impact fibers’ solubility, viscosity, and durability [47]. High-viscosity solutions can form thicker and more uniform fibers, while low-viscosity solutions produce thinner, more hairy fibers [48]. The pH value of the solution can change the polymer’s structure and the solvent’s effect. The solution’s pH changes can affect the fibers’ morphology [49].
- *Electrical parameters* are affected by the applied voltage, collector distance, and electrical field intensity. High voltage draws the solution from the liquid in fine fibers in electrospinning. Voltage affects the diameter and morphology of the fiber [50]. Usually, a voltage between 10 and 30 kV is applied [51]. The distance between the solution droplet and the collector constitutes another crucial design parameter [52]. According to the applied voltage, finer and more uniform fibers can be obtained when this distance is more prolonged. The intensity of the electric field allows the solution to be drawn quickly, which in turn affects the size and structure of the fiber [53].
- *Temperature and humidity constitute the environmental parameters* of this process. Temperature determines the fibers’ properties by influencing the solution’s evaporation rate. The high temperature can help the solvent evaporate quickly, allowing the fibers to form more rapidly [54]. The humidity of the environment can also affect the electrospinning process. High humidity can reduce the rate at which the solvent evaporates and affect the smoothness of the fibers [55].
- *The flow rate and the collector’s properties* determine the parameters related to system design and its applications. The solution’s flow rate directly affects the fibers’ thickness and shape [56]. A high flow rate produces thicker fibers, while a low flow rate produces thinner fibers [57]. The type of collector surface (flat, 3D structures, etc.) affects the arrangement of the fibers. 3D structures can make the fibers settle more smoothly and in a specific order [58]. The movement of the collector surface can change the orientation and arrangement of the fibers [59].

Accurately optimizing these parameters is major in producing nanofibers with the desired properties [43]. Since each parameter affects the other, evaluating more than one parameter together is necessary.

### 3.2. Materials Used and Their Biomedical Applications

The selection of polymers depends on the intended application (e.g., medical, textile, filtration, etc.) and the desired fiber properties (e.g., durability, biocompatibility, surface properties) [60]. Polymers in nanofibers produced by electrospinning for biomedical applications must generally be biocompatible, biodegradable, and have properties promoting cell interaction [61]. The electrospinning technique plays an important role, especially in tissue engineering [62], drug delivery systems [63], wound healing [64], antibacterial coatings [65], and the production of nanofibers for use in biomedical devices [66]. Polymers commonly used in biomedical applications include:

- *Poly (lactic acid) (PLA)* is biodegradable and biocompatible. It has high mechanical strength. Tissue engineering, wound dressings, and biomedical devices (e.g., suture threads) are the primary areas of use. PLA is a biodegradable polymer metabolized in the body over time, producing acetic acid. Due to this feature, it is very suitable for medical implants and biomedical applications. It also has favorable surface properties that promote cell growth [67].
- *Poly (lactic acid-glycolic acid) (PLGA)* is biodegradable, has biocompatible properties, and has a modifiable degradation rate. They are used in tissue engineering, drug delivery, wound healing, and biomedical implants. PLGA is biodegradable and dissolves slowly in the body. These properties make it ideal for long-term drug release and tissue engineering applications. In addition, it is biocompatible and biodegradable, so it does not harm the body [68].
- *Polyvinyl alcohol (PVA)* is water-soluble, biocompatible, and biodegradable. It is used in wound dressings, biotechnological applications, cell culture, and drug delivery. Due to its high biocompatibility, it is suitable for wound healing and cell interactions. It is also widely used in preparing hydrogels for biomedical applications [69].
- *Collagen* is a biocompatible and biologically active natural biopolymer. They are used in tissue engineering, wound healing, biological implants, and cell culture studies. Collagen is a protein that occurs naturally in the body, has a very high biological compatibility, and provides a suitable environment for cells to attach and multiply. It is used in tissue engineering, especially in the repair of structures such as skin, bone, and cartilage [70].
- *Chitosan (Chitin Derivatives)* is natural, biodegradable, biocompatible and antibacterial. Antimicrobial coatings are used in wound healing, tissue engineering, and drug delivery studies. Since Chitosan is a natural polymer with antibacterial properties, it is suitable for wound dressings and biomedical coatings due to its biodegradability and biocompatibility. It promotes cell growth and is used safely in biomedical applications [71].
- *Polyurethane (PU)* shows high flexibility, biocompatibility, and durability. It is used in tissue engineering, wound dressings, biomedical implants, and biological release systems. Its flexible and biocompatible properties make polyurethane ideal for medical devices and biomedical implants [72].
- *Polyacrylonitrile (PAN)* has high mechanical strength, is soluble with suitable solvents, and is biocompatible. It is used in drug transport, tissue engineering, carbon fiber production, and biotechnological applications. PAN has properties that are ideal for electrospinning and forming fibers with high mechanical strength. This feature is especially prominent in tissue engineering applications [73].
- *Hydrogel polymers (Acrylamide, Polyvinyl Pyrrolidone-PVP*, etc.) *are biocompatible materials* with a high water-holding capacity. It has soft and flexible structural features. It is used in cell culture, wound dressings, biomedical coatings, and drug delivery. Hydrogel polymers are particularly suitable for the growth and interactions of cells in water-based media. It has properties ideal for wound healing and tissue engineering [74].
- *Elastin and Elastin Derivatives* have flexible, biocompatible, and tissue-compatible properties. It is used in tissue engineering, especially in elastic tissue repair (e.g., blood vessels, lungs). Since it is a natural protein found in the elastic tissues of the body, it has high biocompatibility and flexibility [75].
- *Silk Fibroin (Silk Fibers)* is a natural polymer that is biocompatible and biodegradable. Structurally, it has high mechanical strength. It is used in tissue engineering, wound dressings, and biotechnological applications [76].

These polymers are the basic nanofiber materials produced by the electrospinning method used in biomedical applications. Polymer selection is made according to the targeted application and requirements, considering biocompatibility, biodegradability, mechanical properties, and cell interaction.

### 3.3. Output Generation Features and Constraints

Electrospinning is a widely used technique for producing fine nanofibers. This technique allows the solution to be drawn in an electrical field to turn it into fine fibers. Electro-spinning is mainly used in biomedical, textile, filtration, energy storage, and environmental applications. However, the electrospinning method has several output features and some limitations.

Electrospinning is very effective for producing fine fibers at the nanometer level, with its capacity to enable the production of nanostructured fibers. These fibers have a very high surface area-to-body ratio. The physical properties of these fibers, with diameters ranging between 10 nm and a few micrometers, can be finely adjusted. The electrospinning technique benefits from a wide range of polymers, which can be biocompatible, biodegradable, flexible, or rigid. Each specific polymer produces nanofibers suitable for particular applications. In addition, different solvents can be used for optimizing the polymer’s solubility and electrospinning performance. The electrospinning method allows different structural arrangements for the fibers to be generated. The fibers may be smooth, uneven, or crosslinked. This flexibility will enable fibers to promote cell interaction, especially in applications such as tissue engineering. Due to these properties, the resulting nanofibers have a large surface area and can provide high coating, filtration, or biological interaction. This property is critical in drug handling, tissue engineering, and biomedical applications.

Electrospinning is a technique that allows large quantities of nanofibers to be produced at a low cost. This makes it attractive for commercial production. Efficient production can be achieved, especially at the laboratory scale. The electro-spinning process can be run at high speed with appropriate parameters to suit large-scale production. However, increasing this speed can adversely affect the quality of the fibers. Although electro-spinning can produce high-quality nanofibers, the total production amount is limited. This can create a constraint, especially in industrial applications that require large-scale production. High production efficiency is difficult to achieve because a low flow rate and high speed are necessary for nanofiber production, but it is challenging to balance both parameters.

During electrospinning, fibers often show irregular or random orientation. This can make achieving the regular and parallel fiber structures required for a particular application challenging. The arrangement of fibers can be essential for producing materials with specific properties. Still, additional processing steps (e.g., the unique design of the collector surface) may be required to ensure proper fiber orientation. Fiber’s surface smoothness and thickness are another limitation of this production method. The surface of nanofibers can often be rough. Some applications require fibers with smoother surfaces, while electrospinning can struggle to control such properties. The non-homogeneous thicknesses of the fibers affect the physical properties of the fibers. The viscosity, solvent, and other properties of the solution can affect the quality of the nanofibers. It is essential to set these parameters correctly, and the solution preparation can sometimes be complicated. Solvent selection may be challenging due to solvents’ toxicity, which requires the consideration of environmental influences.

The collector surface’s shape, size, and rotation speed in the electrospinning process affect the fibers’ arrangement. Different collector surfaces may be required depending on the applications, which can complicate the production process. Using a high-speed collector surface can adversely affect the smoothness and thickness of the fibers. The electrical parameters used in electrospinning, such as voltage, flow rate, and distance, demand careful optimization. Incorrect parameters can prevent the fibers from forming correctly or cause the fibers to come out in undesirable shapes (for example, droplets or bubbles). The evaporation rate of the solvent can pose a problem during the electrospinning process. Rapid evaporation can prevent the fibers from achieving the desired properties because the fibers tend to shrink and thin more. The electrospinning process can be affected by ambient conditions (temperature, humidity). In particular, moisture can change the solution’s viscosity and disrupt the fibers’ homogeneity. This creates a production process that is sensitive to environmental factors.

Electrospinning is an excellent method for producing high-quality and fine nanofibers, but the limitations and difficulties mentioned above can be encountered. To achieve goals such as increasing production speed, controlling the orientation of fibers, and reducing costs, we come across modifications obtained using hybrid 3D production techniques.

## 4. MEW as a Hybrid 3D Manufacturing Technique

### 4.1. Combining Fused Deposition Modelling (FDM) and Electro-Spinning (ES) Techniques

**Figure 3:**
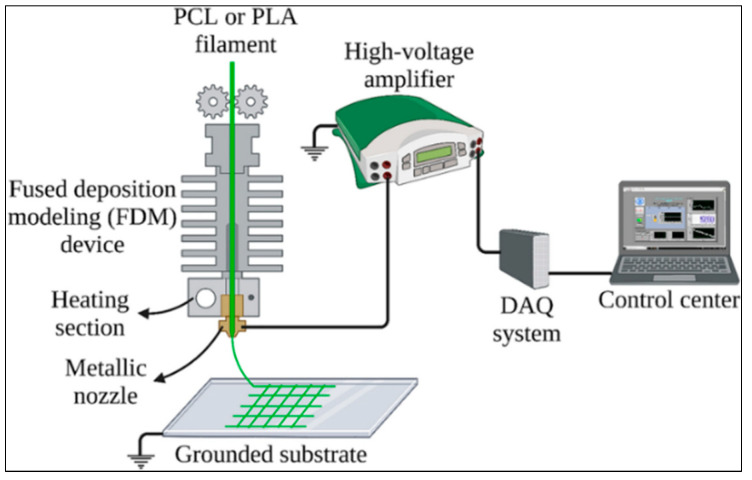
A schematic diagram showing the combined processes of FDM and ES (from [80]).

In recent years, tissue engineering and biomaterial production have shown significant development in biotechnology and medicine. Advances in this field contribute to developing innovative treatment modalities and biomedical devices [77,78]. Scaffold structures used for cellular growth and tissue regeneration play a critical role in tissue regeneration by providing the appropriate environment for cells to adhere, multiply, and differentiate [79]. In this context, advanced production techniques developed for biomaterial production are essential in obtaining suitable scaffolding structures. It is possible to eliminate the disadvantages of FDM and ES using the MEW approach, which combines FDM and ES techniques for the bio-printing process (Figure 3).

The MEW technique, known as the bioprinting method, emerged through integrating Fused Deposition Modeling (FDM) and Electrospinning (ES) techniques and has paved the way for developing high-resolution and cost-effective structures in biomaterial production. FDM is a low-cost and accessible 3D printing technique widely used in producing macroscale structures through the layer-by-layer deposition of polymers [81]. This technology makes it possible to create structures at the macro scale quickly but has limitations in creating detailed structures at the micro and nanoscale [82]. On the other hand, ES allows printing thin polymer fibers at high resolution to obtain scaffold structures at the micro and nanoscale [83]. However, it can be difficult for the fibers to settle in an orderly manner during electrospinning. Fibers are often randomly oriented, which can be problematic, especially for desired regular structures [84]. Creating complex 3D structures with electrospinning is usually challenging, as the production of high-resolution and complex structures often requires additive manufacturing processes [85].

### 4.2. Working Principle and Processing Steps of Melt Electro Writing (MEW) Technique

The MEW process requires a subtle integration of several discrete sub-processing steps:

- *Polymer Selection and Heating:* The MEW method usually works with thermoplastic polymers. These polymers are materials that can melt when heated and have a high viscosity. Polymers melt, usually at high temperatures, so they become liquid and have a consistency suitable for printing. Commonly used polymers include PEEK (Polyetheretherketone), PLA (Polylactic Acid), and PCL (Polycaprolactone).
- *Feeding of the Melt Polymer:* The polymer is melted with the help of a heater and then fed through a thin tip (nozzle). The molten polymer is kept at a specific temperature, ensuring high viscosity and making it suitable for creating fine fibers.
- *Attraction by Electrostatic Force:* MEW uses electrostatic forces, as in the electrospinning method. The molten polymer is passed through a nozzle to which a high voltage is applied, creating an electrical field. The electric field causes the molten polymer to be pulled like a thin thread. At this stage, the high viscosity of the polymer helps form the fiber more controlled and distinctly.
- *Layer-by-Layer Stacked Printing:* The thin polymer fibers are drawn and printed on a specific surface by the movement of the print head. This process takes place layer by layer. Printed with high resolution and precision, the fibers are stacked according to the shape of the predetermined 3D model. Printing the polymer at a low speed while molten ensures the layers are placed correctly. The placement of fibers can be regulated in such a way as to create an environment that promotes the growth of cells, especially in tissue engineering and biotechnological applications.
- *Cooling and Solidification:* The molten polymer fibers cool and solidify rapidly after printing on the surface. This process allows strong bonds to form between the layers and ensures the structural integrity of the printed object. The cooling of the polymer determines the shape and structure of the fiber. The bonds between the layers become stronger during solidification.
- *Formation of 3D Structures:* At the end of the layer-by-layer printing process, the desired 3D structure is obtained. The fibers’ orientation, solubility, and correct placement determine the object’s mechanical properties and biological compatibility. MEW enables the production of materials that support complex structures and multifaceted cellular interactions, especially with dual-axis and three-dimensional printing processes.

A modification-based 3D printer platform design to be made in this direction leads to advantageous printing features such as [86]:

- *High Resolution and Sensitivity: The* MEW technique can produce particularly fine fibers and provide high resolution. This is very important for applications such as tissue engineering because thin and uniform structures are required for the cells to settle correctly.
- *Suitable for Biomedical Applications:* MEW can work with biocompatible and biodegradable polymers, making it ideal for tissue engineering and biological implants. Structures that promote the growth and development of cells can be created.
- *The High Mechanical Strength* of the structures manufactured with MEW is significant for biomedical implants and engineering applications.
- *Compliance with the Structures’ Complexity:* The high resolution makes creating complex and detailed structures with MEW possible. The arrangement and orientation of the fibers can be customized to design specific structures.

Traditionally, obtaining structures with both macro and micro properties in the production of tissue engineering scaffolds requires using different production techniques together [12,79]. In this direction, it is investigated how higher resolution and functional scaffolding structures can be produced cost-effectively by integrating FDM and ES techniques. Theoretically combining ES techniques using a modified FDM platform can allow for the creation of complex geometric structures, as well as make the biomaterial manufacturing process more cost-effective and accessible [87,88].

A key advantage of this integration is that macro and micro features can be combined on the same production platform. With the rapid writing of macro structures with FDM and the addition of micro-level precise details with ES, it is possible to develop multi-scale biomaterial structures [89,90]. Furthermore, the porosity of the scaffolds produced by this method can increase usability in tissue engineering by providing an environment suitable for cell penetration and tissue formation [91,92]. This paper researches what steps should be included in designing a specially modified 3D bio-printing platform suitable for MEW technology, which combines FDM and ES techniques. Possible biomaterials that can be used on such a platform are investigated and listed, along with their properties.

### 4.3. Design Components of MEW Platforms

**Figure 4:**
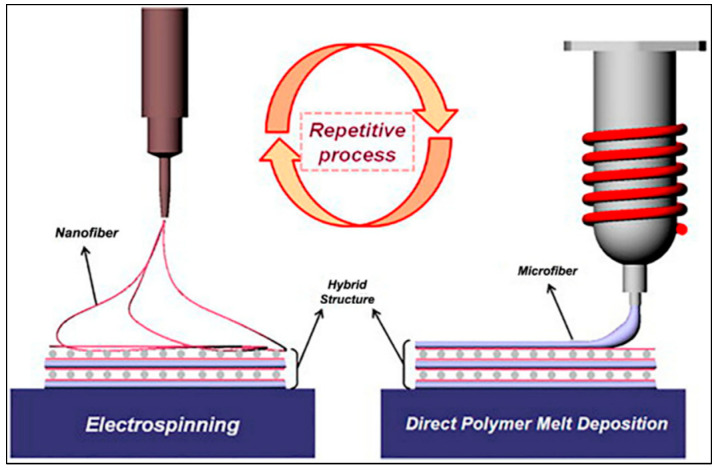
A schematic diagram comparing ES and MEW techniques (from [93]).

In a three-axis cartesian FDM printer, the print head must first be modified to be fed with high-viscosity polymers. Applying a voltage difference with a high-voltage source should ensure that the polymers are laid on the plate in fibers with the tension difference between the nozzle and the table. With this process, it will be ensured that the printer can create the desired mesh structures, thanks to the movable x-y axes and the fixed z-axis. The mixed system envisaged in this direction is structurally and functionally similar to the ES method. Compared to the electrospinning method (Figure 4), the mixed production system created by the integration of Mel Electrowriting (MEW) and Fused Deposition Modeling (FDM) systems offers several advantages in terms of biomaterial production and tissue engineering applications.

The comparison of these technologies and the benefits offered by the MEW system are discussed below.

### 4.4. Modification and Redesigning Steps

Redesigning steps of a bio-printing platform to be modified by integrating FDM and MEW technologies can be identified as follows:

- *Printer Hardware:* Integrating an MEW system onto a standard FDM printer first requires an electric field applicator mechanism to be added to the extruder system of the FDM printer, making it possible to produce both macro and microstructures with the same printer [12].
- *Software Modification:* FDM and MEW writing processes must be executed sequentially or in parallel to obtain specific patterns and geometries [90]. Thus, an algorithm is needed to program the sequential FDM and MEW processes.
- *Printer Consumables:* The basic materials used in the printing phase should be selected from thermoplastic polymers suitable for Fused Deposition Modelling (FDM) and Melt Electro Writing (MEW) technologies. For example, Polycaprolactone (PCL) is a biodegradable and biocompatible polymer widely used for tissue engineering [94]. It is often preferred in FDM and MEW printers. Polylactic acid (PLA) is a biodegradable polymer that is desired to form macrostructures for the FDM process [95].
- *Reactive Materials:* If surface modifications are to be made for cell culture studies, chemical reagents may need to be used. For this purpose, it is possible to improve cell adhesion properties by using biomolecules such as collagen or fibronectin for surface modifications [79,96].

The modifications required for integrating the FDM printer with the MEW technology aim to combine the working principles of the FDM system with the fine fiber production capability of the MEW. The modifications that need to be made for this purpose can be phased as follows:

- *High Voltage Electric Field System Integration:* MEW uses high voltage to enable the polymer to be printed into fine fibers while in the molten state. Therefore, a power supply that generates high voltage must be integrated into the FDM printer. The power supply should be installed in an area close to the printer’s extruder and properly positioned to allow electrical withdrawal of the polymer.
- *High Voltage Usage* for MEW applications generally ranges from 5 to 30 kV. For this purpose, a power supply is required to produce low current but high voltage capacity. The voltage range should be adjustable according to the material used and the diameter of the fibers to be generated. The power supply must have a stable and adjustable voltage output. The high-voltage source is used to pull the polymer in a controlled manner and ensure smooth fiber production. The accuracy of the high voltage can directly affect the quality of the fibers to be produced.
- *Flow Mechanism of the Melt in MEW:* MEW ensures the flow of the melt polymer through the nozzle using electro-hydrodynamic force. By applying a high voltage of 10–30 kV, the polymer melt is drawn towards a target surface grounded in fine fibers [97]. In this process, the surface tension on the melt is broken by the effect of the electric field, and thus, very fine fibers are obtained. The high voltage used in this method allows the polymer to flow in a controlled manner and to form fibers with micrometric diameters [95].
- *Metal Grounding Plate:* The MEW system uses a metal grounding plate to collect the polymer fibers properly. It is possible to modify the existing print table of the FDM printer with a conductive material. The grounding of this metal table is critical for the controlled printing of the polymer.
- *Modification of the Nozzle:* The nozzle used in the MEW system has a structure different from that of the FDM nozzle, which allows the polymer to be extruded into fine fibers. Therefore, the FDM printer’s nozzle should be replaced with a smaller diameter for high-temperature control. In addition, the nozzle will need to be insulated and made suitable for high voltage. This must be done to prevent leakage current from occurring when high voltage is applied to the nozzle.
- *Adaptation of Printer’s Movement Mechanism and Control System:* High precision is required on the FDM printer’s moving x-y-z axes. Since micro-scale production will be made with the MEW method, it may be necessary to increase the movement speed and accuracy of the printer.
- *X-Y-Z Axis Motion Precision in FDM* generally has an accuracy of 0.1 to 0.2 mm, which is considered sufficient for desktop printers. However, high-precision industrial FDM printers can operate at a resolution of 50 microns (0.05 mm) or less.
- *Precision Required for MEW:* A much more precise positioning system is required compared to an FDM printer. In MEW, exact x-y-z axis control is used to achieve a resolution of 1–10 microns [98]. This precision is essential for the smooth placement of fibers with high resolution and micrometric size. To achieve this precision, MEW printers use precision motion control systems, such as piezoelectric or linear motors [99]. It is possible to use the existing control system of the FDM printer. Still, based on the printing results obtained, planning a control circuit modification compatible with the MEW process is necessary. This is significant for the nozzle’s movement speed and the high voltage to be controlled in synchronization.
- *Temperature control is essential so* that the polymer used for MEW can be in a molten state and have the appropriate viscosity. Therefore, it is crucial that the temperature control unit of the extruder is precise and that the polymer can be kept at the correct temperature. The nozzle temperatures required in FDM vary depending on the material used. For example, PLA (Polylactic Acid) is in a typical range of 180–220 °C [100]. PLA is preferred due to its low melting temperature and good fluidity properties. ABS (Acrylonitrile Butadiene Styrene) is used at a temperature of 210–250 °C and has advantages in higher temperature demanding processes [101].
- *The polymers used in MEW* need to be melted at precise temperatures for viscosity control. Materials such as PCL (Polycaprolactone) melt between 60–90 °C, and the appropriate viscosity is obtained at this temperature [102]. This temperature range enables the production of high-resolution fibers and supports the flow of the polymer under an electric field. The existing temperature control system of the FDM printer must be modified to match the temperature sensitivity to ensure both the fluidity and electrical attraction of the polymers in MEW.

### 4.5. Comparison of ES and MEW Techniques

**Figure 5:**
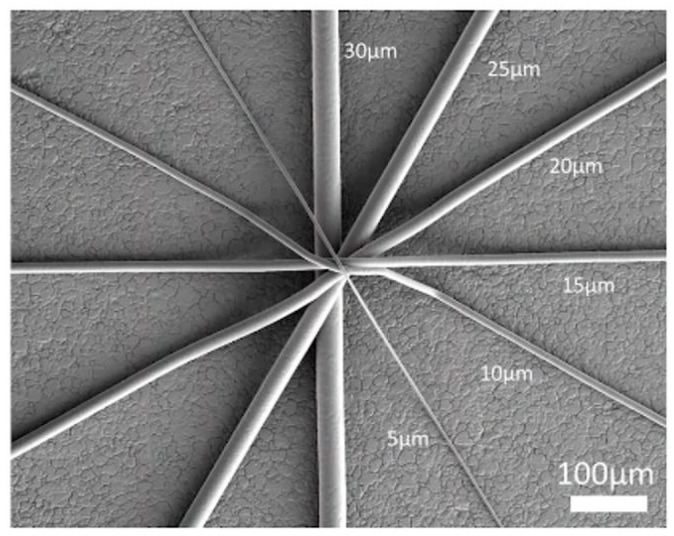
Fiber Sizes: Thinner fibers can be produced by the MEW method (from [105]).

Electrospinning is a technique to generate fine fibers from polymer solutions or melts under an applied electric field. It successfully forms nano- and micro-scale fibers, thus providing a suitable surface for the adhesion and proliferation of cells [103]. However, the fibers produced by the electrospinning method are irregular, which makes it difficult to control certain structural features [104]. However, MEW works on a similar principle to electrospinning; it makes a difference by writing the polymer in a molten state and arranging the fine fibers in a more controlled way (Figure 5) [105]. Compared to electrospinning, MEW provides a higher resolution and repeatable structural layout because it uses a system that can control flow rate and pressure. In addition, thanks to the direct writing technique, the positioning of the fibers is done precisely, which allows the creation of regular structures. Using a high-viscosity melt in MEW makes it easier for the fibers to maintain the desired geometric shape, reducing the irregularity encountered in electrospinning [92,106]. Therefore, structures produced with MEW can be fabricated in specific patterns and geometric layouts, offering a more favorable scaffold for cellular growth [83].

#### 4.5.1. Structural and Mechanical Differences

**Figure 6:**
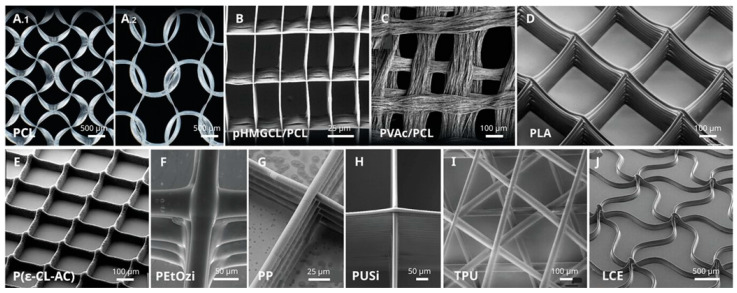
Examples of SEM images of different scaffolding structures printed with MEW (from [108]). (**A**) Polycaprolactone (PCL). (**B**) PCL/poly(hydroxymethylglycolide-co-ε-caprolactone) (pHMGCL) blends. (**C**) PCL/poly(vinylacetate) (PVAc) blends after PVAc dissolution. (**D**) poly(lactic acid) (PLA). (**E**) poly(ε-caprolactone-co-acryloyl carbonate) p(ε-CL-AC). (**F**) poly(2-ethyl-2-oxazine) (PEtOzi). (**G**) polypropylene (PP). (**H**) poly(urea-siloxane) (PUSi). (**I**) thermoplastic polyurethane (TPU). (**J**) liquid crystal elastomers (LCE).

The fibers produced by electrospinning often have a random distribution, and it isn’t easy to establish a desired geometric order [107]. This can be limiting for applications such as specific mechanical properties or the orientation of cells in a particular direction. On the other hand, since the fibers produced with MEW offer the possibility of forming a controlled pattern (Figure 6), higher control over mechanical properties and cellular organization is achieved [12,108].

The hybrid system, designed by integrating FDM and MEW, makes producing macro and micro-scale structures possible, allowing multi-scale structures that cannot be achieved by electrospinning. This allows for the combination of properties such as both macroporosity and cell adhesion with microstructures in tissue engineering applications [109].

#### 4.5.2. Advanced Scaffolding Design for Tissue Engineering

**Figure 7:**
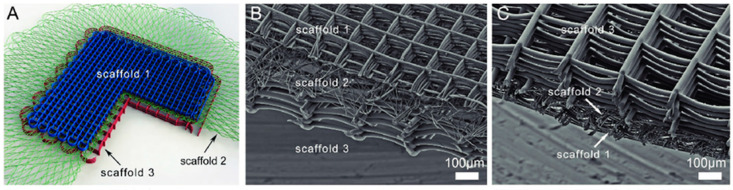
Examples of scaffolding structures generated by MEW (from [105]). (**A**) A schematic of a dual-sized scaffold design, with a middle layer of small diameter fibers (4 µm) to improve cell seeding efficiency. Image of such a trilayered scaffold visualizing (**B**) scaffold 1 (10 µm diameter fibers; 125 µm spacing) and (**C**) scaffold 3 (25 µm diameter fibers; 250 µm spacing) from above.

Tissue engineering involves using scaffold structures made of biomaterials to regenerate damaged or dysfunctional tissues within the body [110]. The scaffolds produced for this purpose must be designed to provide a suitable environment for cell adhesion, proliferation, and differentiation [79]. The system created by integrating FDM and MEW makes it possible to produce scaffolds with macro and micro-scale structures (Figure 7) [105] in a single process. These multiscale structures have been shown to meet requirements such as macroporosity and microtissue organization in cellular growth and tissue regeneration [111].

**Figure 8:**
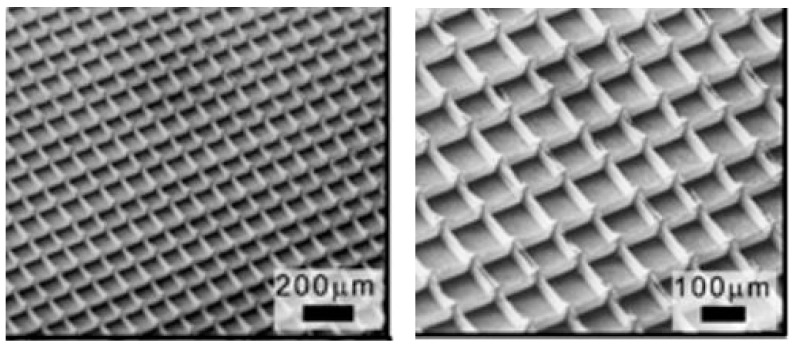
Examples of lattice structures of MEW scaffolds (from [92]).

Studies in the literature show that MEW technology enables cells to be organized in a particular order, thanks to its capacity to form controlled microstructures (Figure 8) [95]. By integrating FDM and MEW, these microstructures can be added to macroporous scaffolds to obtain more complex tissue structures and create an ideal environment for cellular growth [90]. This allows particularly complex tissues, such as cartilage or vessel-like structures, to be engineered more efficiently.

#### 4.5.3. Differences of Polymers Used in MEW

There are several differences between the polymers used in terms of:

*Electrical Properties:* The polymers used in MEW must have a specific dielectric property to be driven by electrostatic forces. Therefore, the polymer’s electrical conductivity properties directly affect the efficiency of the process [105].

*Ionization State:* The ionization of the polymer in MEW strengthens its interaction with the electric field, which ensures that the polymer fibers are correctly oriented.

*Additional Solution Requirement:* In some cases, solvents may be added to improve the flow properties of the polymer. For example, additional substances can adjust viscosity or inhibit the electrospray effect [112].

*Polymer Feed and Flow Characteristics of the Melt:* The syringe pump plays a critical role in precisely feeding the polymer used in MEW processes. 0.1–5 μL/min is a suitable range for a feed rate [112]. This range may vary depending on the material and targeted fiber thickness. Polymer flow rate is related to parameters such as fiber diameter and viscosity to be obtained. For example, a higher feed rate is used for thicker fibers, while lower feed rates are used for thinner fibers [113].

#### 4.5.4. Cellular Growth and Biocompatibility

Since the fibers produced by electrospinning can be at the nanoscale, they provide an advantage in terms of the capacity of the cells to adhere to the surface and migrate. However, the random distribution of these fibers can make it difficult for cells to organize in a controlled manner [114]. The MEW system can produce controlled microstructures that allow cells to be organized in a specific direction, allowing the cells to grow in a way that is more suited to the targeted tissue [12]. Multi-scale scaffolds produced with the integration of FDM and MEW combine the advantages of two different structures in terms of cellular growth. Macrostructures created with FDM let cells penetrate deeper parts, while the microstructures added with MEW allow better cell adhesion to the surface [115].

Productions made by solution-based methods such as electrospinning can have adverse effects on biological systems due to solvent residues. This creates a disadvantage regarding the biocompatibility of the biomaterials produced [116]. Structures generated with FDM and MEW integration have a more significant advantage in biocompatibility and safety, as they do not require solvents [79]. Melted polymers eliminate solvent use and thus remove related chemical residues, making biomaterials safer and suitable for direct clinical applications. It has been stated that multi-scale structures printed with FDM and MEW technologies are created using biocompatible polycaprolactone (PCL) material. These structures provide an ideal environment for the adhesion and proliferation of cells [90]. This is an essential step in developing biomaterials that can be used in clinical applications.

#### 4.5.5. Efficiency and Cost-Effective Biomaterial Production

Although electrospinning allows fine fibers to be produced quickly to cover large areas, it may require more processing since the structures created are not in a specific form and regular structure, which can reduce productivity. In addition, solution preparation and solvent removal complicate the process and increase the cost. Traditional methods for the production of biomaterials often require costly and complex devices. Bio-printers, especially those used to obtain high-resolution structures, are expensive and usually not feasible on a laboratory scale. Integrating MEW and FDM technologies offers a cost-effective solution by modifying commonly used FDM printers with MEW technology. This could enable more laboratories in the field of biomedical engineering to access these technologies, speeding up research and development processes [115].

Integrating the high-voltage system used in the MEW process with FDM printers suggests a robust and cost-effective manufacturing method [92]. This modification makes biomaterial production accessible on a large scale while offering a less complex and lower-cost way to produce high-quality scaffolds. In this way, a more expansive production and testing capacity can be achieved for tissue engineering applications in biomedical engineering.

#### 4.5.6. Production Process and Controllability

Since the fibers produced by electrospinning are usually solution-based, they require solvents, and the residues of these solvents can have adverse effects on biological systems [79]. This can lead to potential biocompatibility issues in biomedical applications. On the other hand, FDM methods integrated with MEW eliminate solvent usage by involving molten polymers, providing a safer environment for biological systems. In electrospinning, environmental factors such as temperature and humidity significantly impact the formation of fibers, and these factors are difficult to control [115]. In contrast, in molten polymer-based methods such as MEW and FDM, the influence of such environmental factors is less, and the reproducibility of the production process is higher [117].

As a result, the MEW technique, as a hybrid system created by combining FDM and ES techniques, has advantages such as more excellent controllability, no use of solvents, and more suitable microstructures in terms of cell adhesion and growth compared to electrospinning. This integration enables the production of high-resolution and cost-effective scaffolds for tissue engineering and biomaterial production, offering a better alternative in terms of efficiency and quality compared to the electrospinning method.

## 5. Conclusions

Designing an MEW platform by combining FDM and ES methods [112] and modifying FDM printers with the MEW system [118] makes it possible to develop bioprinter platforms by integrating standard FDM printers with MEW. Micron-diameter fibers can be produced from biomedical polymers such as polycaprolactone (PCL) using low-current, high-voltage sources. These fibers have properties suitable for tissue engineering applications [119]. Again, such an integration allows the production of high-resolution scaffolding structures with adjustable fiber quality properties [86]. This hybridized manufacturing technique paves the way for developing more accessible and flexible production platforms for research in regenerative medicine, pharmacy, and biomedical fields, especially tissue engineering applications [102].

Since the MEW method requires the polymers used to melt at high temperatures, MEW can only work with thermoplastic polymers. The layer-by-layer production process slows production time, resulting in a challenge to yield high-volume production. Despite these difficulties and limitations, which are based on the working principle of the hybrid manufacturing technique used, MEW is a powerful and promising technology that produces high-resolution 3D structures by attracting molten polymers with electrostatic force and making controlled prints.

The ability to produce micro and macro structures at the same time offers significant advantages in terms of cellular growth and tissue regeneration. This method is promising, especially for reconstructing complex tissues such as nerves, muscles, and vessels. The development of multiscale scaffolds allows cells to attach, multiply, and differentiate more effectively. This increases the success rates in tissue engineering and offers innovative solutions for complex tissue damage treatment.

With all these features, the FDM-ES modified MEW technique is a practical manufacturing platform with promising research, development, and application opportunities, allowing the creation of structurally complex, finer, and unique fibers, especially in tissue engineering and biotechnological applications.

## Data Availability

No new data were created or analyzed in this study. Data sharing is not applicable to this article.
